# Bilateral neck of femur stress fractures in unilateral hip pain: Is magnetic resonance imaging (MRI) of the contralateral hip necessary?

**DOI:** 10.5339/qmj.2026.65

**Published:** 2026-09-15

**Authors:** Vernon Tan Sze Yuen, Faris Indra Prahasta Bin Didi Indra

**Affiliations:** 1 Orthopaedic Department, Faculty of Medicine and Health Sciences, Universiti Malaysia Sarawak, Kota Samarahan, Sarawak, Malaysia

**Keywords:** Stress fracture, femoral neck, bilateral hip, MRI

## Abstract

**Background:**

Stress fractures may occur in healthy individuals following repetitive physical loading. They account for up to 10% of all orthopaedic injuries but rarely involve the femoral neck.

**Case Presentation:**

We are reporting a young adult presenting with unilateral hip pain who was ultimately diagnosed with bilateral femoral neck stress fractures. Magnetic Resonance Imaging (MRI) revealed an occult contralateral fracture not visible on plain radiographs. Bilateral screw fixation was performed, with an excellent outcome at two years.

**Discussion:**

In this patient, stress fractures occurred on the bilateral femoral neck secondary to his intensive training for fire brigade recruitment involving running and repetitive jumping jacks. Although the patient presented with unilateral hip pain, given the history and the nature of his job, it is important to screen the contralateral hip to prevent misdiagnosis.

**Conclusion:**

The patient underwent bilateral neck of femur screw fixation and demonstrated an excellent outcome at 2-year post-operative follow-up. This case emphasizes the need for high suspicion and routine MRI of both hips in similar presentations.

## 1. INTRODUCTION

Stress fractures result from repetitive mechanical loading that overwhelms the remodeling capacity of otherwise normal bone. They account for up to 10% of all orthopaedic injuries, though femoral neck involvement is less common.^1,2^ Bilateral fractures are particularly rare but clinically significant because delayed diagnosis increases the risk of displacement, avascular necrosis, and poor functional outcomes.^1^ Risk factors include age, female gender, lower aerobic fitness, prior inactivity, high training volume such as military or uniformed services training and marching, and smoking.^2^

We report a rare case of bilateral femoral neck stress fractures (FNSF) in a healthy male with unilateral hip pain who presented to a tertiary government hospital emergency department in Malaysia and was treated surgically. Signed informed consent was received from the patient for his case to be used as teaching material and for publication purposes. 

## 2. CASE PRESENTATION

A 25-year-old healthy male presented to the emergency department of a tertiary hospital with right hip pain for two weeks, progressing to inability to bear weight for the preceding two days. He denied preceding trauma or infection-related symptoms. His history revealed intensive training for fire brigade recruitment involving running and repetitive jumping jacks, which commenced 1 month ago. 

On examination, there was tenderness and restricted motion in the right hip. The left hip was asymptomatic and clinically normal. Laboratory investigations, including serum calcium and inflammatory markers, were unremarkable. Baseline anthropometric data showed the patient was 175cm tall and weighed 80kg, with a calculated body mass index (BMI) of 25.4. 

**Figure 1.**
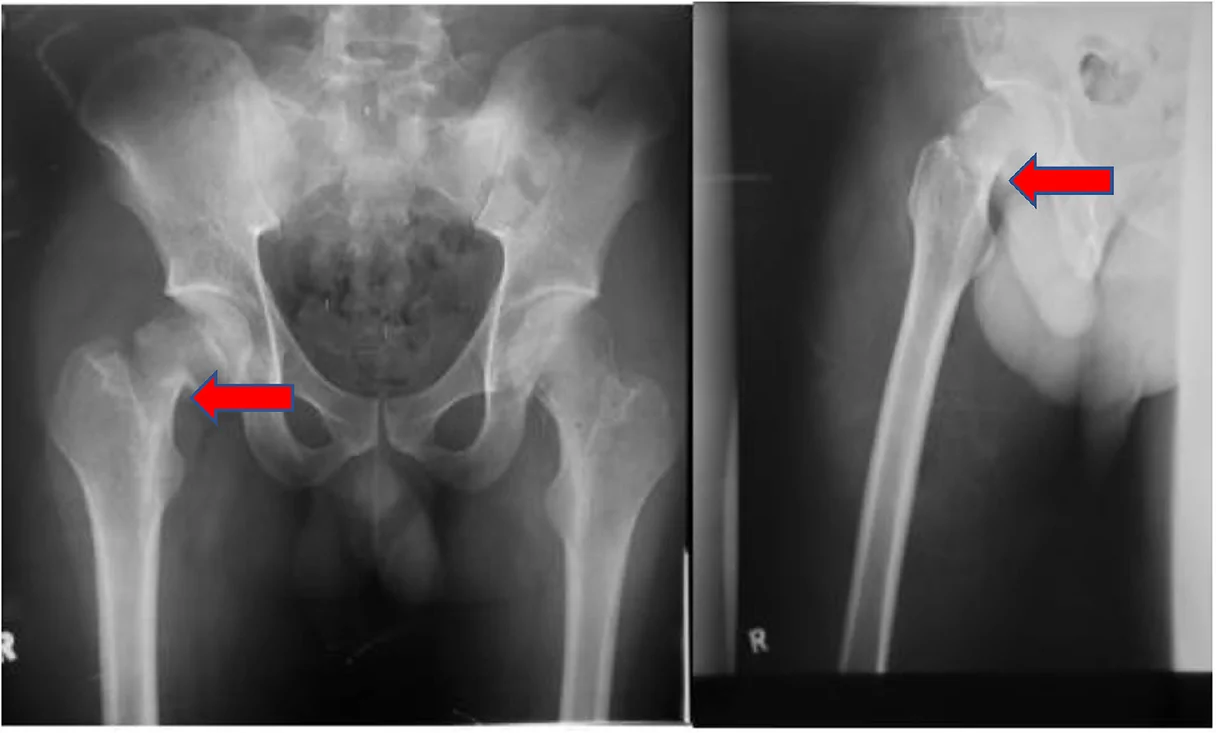
Plain radiograph of pelvis AP and right hip lateral view showing right neck of femur fracture.

**Figure 2.**
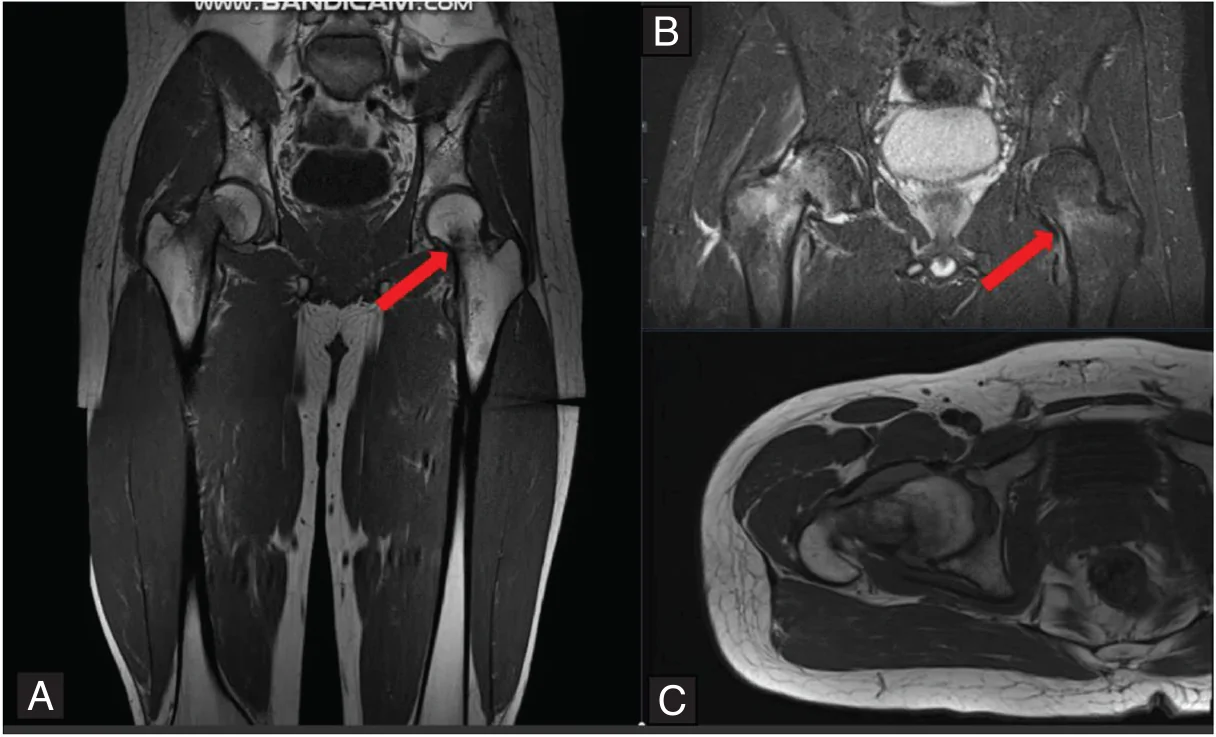
(A) MRI hypointense signal changes over the left neck of femur in T1W image. (B) MRI hyperintense signal changes over the left neck of femur in T2W STIR image. (C) MRI axial cut of right hip showing hypointense signal changes over the neck of femur.

Pelvic radiographs demonstrated a displaced right subcapital femoral neck fracture. The left hip appeared normal radiographically (Figure 1). Given the patient’s history of bilateral stress loading, magnetic resonance imaging (MRI) of both hips was performed. This confirmed a displaced right subcapital fracture and revealed a subtle linear fracture line with surrounding marrow edema in the left femoral neck, despite the absence of clinical symptoms (Figures 2A–C).

**Figure 3.**
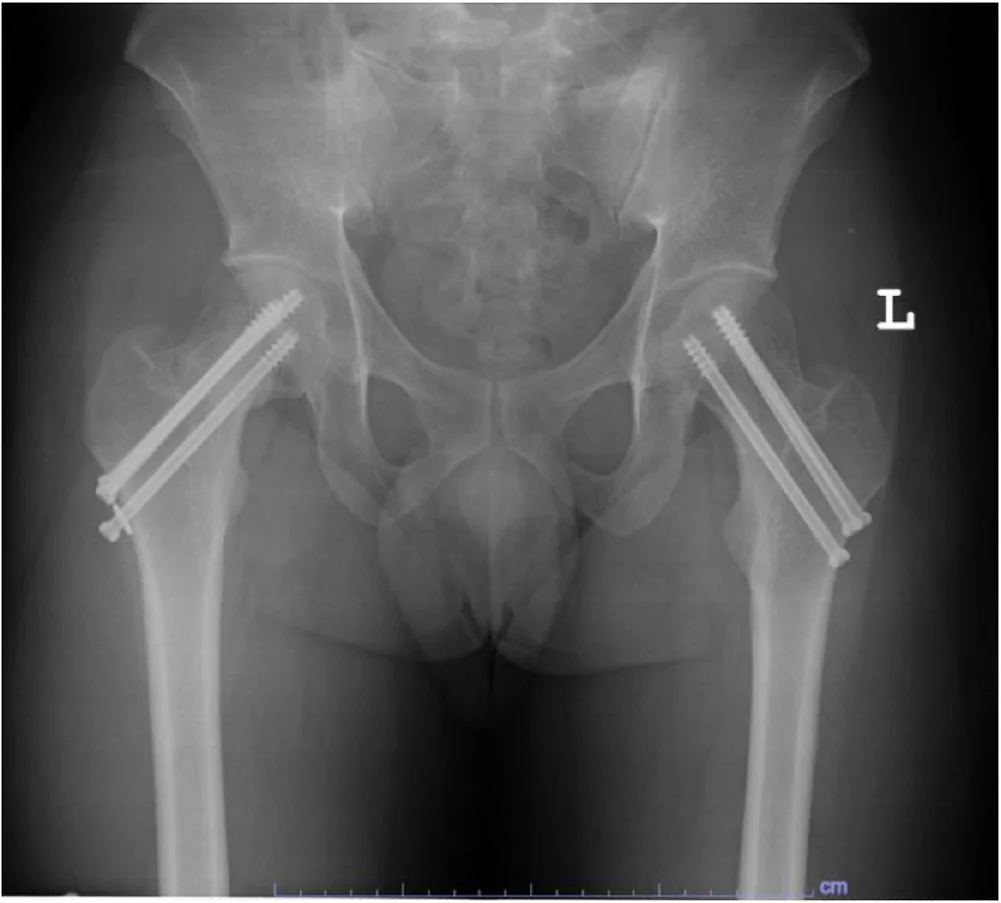
Pelvis AP radiograph showing fracture union over bilateral neck of femur.

The patient underwent bilateral closed reduction and internal fixation using 7.3 mm cannulated screws 2 days after admission. Post-operatively, he was mobilized with gradual progression to full weight by week 10. He started with non-weight-bearing wheelchair ambulation weeks 1-6 post-operatively but kept his bilateral hip range of movement supple. Subsequently, from week 7 onwards he was allowed partial weight bearing and achieved full weight bearing by week 10. Radiographic union of both fractures occurred at three months. At two years, he had returned to full duty as a fireman, with no pain or restriction of hip function (Figure 3).

## 3. DISCUSSION

FNSFs are rare, representing 1–3% of femoral neck fractures and 3–5% of all stress fractures.^2^ If undetected or displaced, they may lead to avascular necrosis and long-term disability.^1^

Stress fractures were first described by Breithaupt in 1855 among Prussian soldiers undergoing repetitive marching.^1^ They occur when cyclical loading exceeds the bone’s adaptive capacity, resulting in cortical microdamage and eventual fracture in non-pathological bone.^1^

Most patients report anterior groin pain exacerbated by activity, though pain may radiate to the thigh or knee.^3^ A common pitfall is misdiagnosis as soft tissue injury, especially because early radiographs are often normal. Only one-third of patients demonstrate abnormalities on initial radiographs, with many remaining negative for 6–8 weeks. Delayed or missed diagnosis can result in propagation and displacement.^3–5^

Plain X-ray may reveal sclerosis, callus formation, or fracture lines, but its sensitivity is limited in femoral neck fracture.^6^ MRI is considered the gold standard due to its high specificity and sensitivity.^7,8^ It can identify marrow edema and subtle fracture lines before radiographic changes are visible. MRI findings typically include hypointense fracture lines on all sequences with surrounding hyperintense edema on T2 or STIR (Short Tau Inversion Recovery) images.

Classification systems guide diagnosis and prognosis. Fullerton and Snowdy^6^ categorized fractures into tension-side, compression-side, and displaced. Provencher et al. later described atypical incomplete tension fractures visible only on MRI.^9^ Shin and Gillingham^10^ further subclassified compression injuries by extent of fracture line across the neck. Arendt and Griffith^11^ proposed an MRI grading system from marrow edema only (grade 1) to visible fracture lines (grade 4) with prognostic implications.

Identifiable risk factors for stress fractures have been discussed in the literature, including male gender, increased height, low bone mineral density (BMD) secondary to old age, and elevated parathyroid hormone levels, particularly among male military personnel.^12^While FNSF is more commonly reported in males, Niva et al.^13^ noted a higher incidence of this injury among female military recruits. Importantly, most symptoms associated with stress fractures in this study tend to be mild and are less likely to progress if left untreated.^13^Additionally, a prior low level of physical activity has been identified as a significant risk factor for stress fractures in recruits undergoing army training.^14^Furthermore, acetabular retroversion has been recognized as a risk factor; however, screening procedures are only recommended for individuals with symptoms.^15^

Management depends on site, displacement, and completeness of the fracture. Displaced, complete tension, and atypical tension fractures require surgical fixation.^16^ Options include multiple cannulated screws or dynamic hip screws. Faster surgical intervention following injury, along with anatomical alignment or valgus reduction of the injury in surgically treated cases, are associated with a reduced incidence of avascular necrosis of the femoral head.^4^ Stable compression fractures may be treated conservatively with restricted weight bearing, though many surgeons prefer permanent screw fixation to avoid displacement.^4^

It is recommended that high-risk individuals, such as runners and army recruits, undergo early screening upon the emergence of symptoms. Furthermore, educational initiatives should be implemented for high-risk groups to enhance awareness of the importance of early diagnosis and timely intervention.^5,11^

Our patient’s contralateral fracture was asymptomatic and radiographically occult but detected by MRI. Without imaging, the fracture could have progressed to displacement, significantly worsening prognosis. Early identification allowed bilateral fixation, resulting in union and excellent functional recovery.

## 4. CONCLUSION

This case underscores the importance of bilateral hip MRI in young, physically active individuals presenting with unilateral hip pain. Even in the absence of contralateral symptoms, bilateral evaluation is crucial to preventing missed injuries. Our patient was treated surgically with bilateral neck of femur screw fixation, which resulted in an excellent outcome at 2-year post-operative follow-up. FNSF, though rare, carry significant morbidity if misdiagnosed or untreated. MRI is essential for accurate diagnosis, particularly for detecting occult contralateral fractures. In active patients with unilateral hip pain, bilateral MRI should be considered mandatory. Early diagnosis and surgical fixation prevent displacement, complications, and long-term disability.

## ACKNOWLEDGEMENT 

The author(s) have no acknowledgements to declare.

## AUTHORS’ CONTRIBUTIONS 

VTSY: Conceptualization, methodology, investigation, data curation, writing – original draft, writing – review & editing. FI: Methodology, formal analysis, investigation, writing – review & editing, supervision. All authors have read and approved the final version of the manuscript.

## CONFLICT OF INTEREST

The authors declare that they have no conflicts of interest related to this case report. 

## DISCLOSURE OF AI USE 

No artificial intelligence tools were used in the preparation, writing, editing, or analysis of this manuscript.

## DATA AVAILABILITY 

This case report is written based on the patient admitted to our hospital as described. 

## FUNDING

The author(s) received no financial support for the research, authorship, and/or publication of this article.

## ETHICAL APPROVAL 

Written informed consent for the publication of clinical details and images was obtained from the patient. The documentation is available with the corresponding author.

## DECLARATION OF PATIENT CONSENT 

The patient has consented for his case to be used as teaching material and publication in Qatar Medical Journal.
